# An efficient scheme for mental task classification utilizing reflection coefficients obtained from autocorrelation function of EEG signal

**DOI:** 10.1007/s40708-017-0073-7

**Published:** 2017-12-09

**Authors:** M. M. Rahman, M. A. Chowdhury, S. A. Fattah

**Affiliations:** Bangladesh University of Engineering and Technology (BUET), Dhaka, 1000 Bangladesh

**Keywords:** Electroencephalogram (EEG), Brain–computer interface (BCI), Autoregressive (AR) model, Autocorrelation function, Reflection coefficient

## Abstract

Classification of different mental tasks using electroencephalogram (EEG) signal plays an imperative part in various brain–computer interface (BCI) applications. In the design of BCI systems, features extracted from lower frequency bands of scalp-recorded EEG signals are generally considered to classify mental tasks and higher frequency bands are mostly ignored as noise. However, in this paper, it is demonstrated that high frequency components of EEG signal can provide accommodating data for enhancing the classification performance of the mental task-based BCI. Instead of using autoregressive (AR) parameters considering AR modeling of EEG data, reflection coefficients obtained from EEG signal are proposed as potential features. From a given frame of EEG data, reflection coefficients are directly extracted by using the autocorrelation values in a recursive fashion, which avoids matrix inversion and computation of AR parameters. Use of reflection coefficients not only provides an effective feature vector for EEG signal classification but also offers very low computational burden. Support vector machine classifier is deployed in leave-one-out cross-validation manner to carry out classification process. Extensive simulation is done on an openly accessible dataset containing five different mental tasks. It is found that the proposed scheme can classify mental tasks with a very high level of accuracy as well as low time complexity in contrast with some of the existing strategies.

## Introduction

Electroencephalogram (EEG) has gained rigorous attention from the researchers for the study of brain–computer interface (BCI). EEG-based BCI systems employ electrical activity of brain to classify different EEG signals corresponding to various mental and motor imagery (MI) tasks correctly. One way to classify the signals effectively is to acquire discriminative features from that signal. As a matter of fact, different schemes to extract distinctive features are available in literature. For instance, in [1], statistical data extracted from cross-correlation of EEG signals are reported as distinctive features for MI task classification, but the main drawback is the usage of prior information of the classes. In order to find frequency bands which can substantially segregate the feature vectors corresponding to two classes of MI tasks, a Bayesian framework is proposed in [2]. However, the method offers moderate classification performance. For the purpose of EEG channel reduction for MI task, various types of spatial filters are widely employed, where regularized parameters need to be chosen manually. To be more specific, in [3], the task of channel reduction is performed by using sparse spatial filter optimization method, where manual intervention is required for obtaining some parameters. In [4], the common spatial pattern (CSP) with generic learning is proposed for EEG channel reduction where optimal selection of regularized parameters needs further investigation. However, apart from MI task classification, several researchers concentrate in various mental task classification. For example, in [5], along with conventional lower spectral bands, an additional band (24–37 Hz) is used to extract spectral power and asymmetry ratio features for mental task classification. This method provides comparatively satisfactory classification performance, but the classification accuracies are not consistent for all cases. In [6], along with the frequency bands used in [5], an additional high frequency band (40–100 Hz) is used in similar feature extraction scheme to obtain better classification performance for mental task. Considering sixth-order autoregressive (AR) system, in [7], AR model coefficients are extracted from given EEG data and used for mental task classification. Moreover, feature extraction scheme based on multivariate AR models are reported in [8], where four different representations of AR coefficients are tested to classify mental task. In [9], along with AR parameters generalized Higuchi fractal dimension spectrum is utilized for mental arithmetic task recognition. In [10], classification of both EEG mental and cognitive tasks is reported based on Wavelet packet entropy and Granger causality, where classification performance is evaluated using multiple kernel learning support vector machine (SVM) classifier. A multivariate feature selection method based on wavelet transform and empirical mode decomposition is proposed in [11] for mental task classification.

One of the main objectives of this paper is to extract robust feature by using autocorrelation function of the EEG signal. For this purpose, reflection coefficients are computed directly from autocorrelation function of the EEG data. The idea of using reflection coefficients as feature is investigated for motor imagery tasks, and some preliminary results are reported in [12, 13]. SVM classifier is used to carry out classification process. The effect of filtering using widely common frequency bands and that of using different kernels is investigated. Simulation details are introduced considering an openly accessible EEG dataset on various mental tasks.

## Data acquisition

A widely used EEG data set collected by Keirn and Aunon [14] is utilized in this paper. EEG signals are acquired from the locations C3, C4, P3, P4, O1, and O2 which are denoted as the $10-20$ international system of electrode placement. Measurements are made considering A1 and A2 as reference. Data are band pass filtered using an analog filter with band limit of $0.1-100$ Hz and sampled at 250 Hz with 12 bit quantizer. The recording is carried out for ten seconds during each session. EEG signals from seven subjects performing five different mental tasks, namely geometrical figure rotation (R), mathematical multiplication (M), mental letter composing (L), visual counting (C), and baseline-resting (B) are investigated. For notational convenience, hereafter, each task is abbreviated with an alphabet as shown in the parentheses. However, data obtained from three subjects contain fewer than ten sessions or have some recording errors. Hence, like some other existing research works [5], in this paper, data from four subjects, each having ten or more sessions, are taken into consideration.

For the purpose of analysis of each ten second session, a number of frames with shorter time interval are investigated as EEG signal is assumed to be non-stationary. In this case, one second frame duration is considered with 0.5 second frame shift (i.e. $50\%$ overlap between successive frames) [6], which provides reasonable number of samples (250 samples) in each frame.

## Proposed method

The proposed method of mental task classification utilizing EEG signal consists of three major steps: preprocessing, feature extraction and classification. In what follows, detailed description of these steps is presented.

**Fig. 1:**
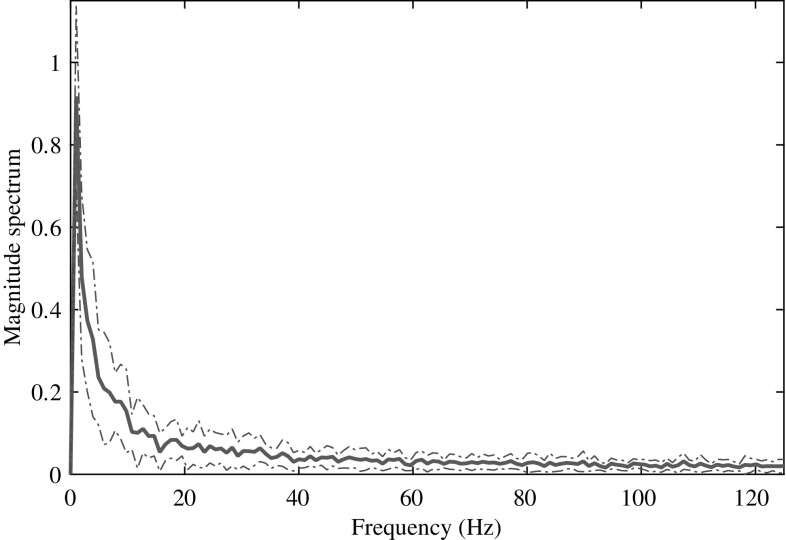
Average magnitude spectrum corresponding to a session of mathematical multiplication task obtained from C3 channel of subject 1. The dotted line on both sides of the average spectrum indicates the standard deviation. (Color figure online)

### Preprocessing

In EEG signal analysis, depending on the nature of practical applications, different well-defined narrow frequency bands , namely delta ($< 4$ Hz), theta (4–7 Hz), alpha (8–13 Hz), beta (14–20 Hz), and gamma (24–37 Hz) are widely investigated for feature extraction [5]. However, in the current application of mental task classification, it may not be useful to restrict the EEG signal analysis only to these low frequency bands. The reason behind is explained as follows. It is well known that while performing mental tasks, relatively high frequency bands (e.g. beta bands or even higher) remain active. Considering this fact in [5], for the purpose of mental task classification, frequency band up to 37 Hz and in [6], frequency band up to 100 Hz are used. In view of investigating the presence of high frequency components in EEG signal during mental tasks, spectral analysis on a large number of EEG frames taken from different channels is carried out. In Fig. 1, average values of magnitude spectra along with standard deviations, computed from 19 consecutive overlapping frames of EEG signal obtained from subject 1 considering mathematical multiplication task, are plotted. As mentioned before, these frames correspond to one complete session within which the mental task is performed. It is clearly observed from this figure that substantial amount of spectral information exists in high frequency region ($>40$ Hz) of the averaged magnitude spectra. It is found that the patterns of the averaged spectrum obtained in different other sessions exhibit quite similar nature. As a result, in the proposed method, the whole frequency band of EEG signal is utilized in order to extract spectral information residing in higher frequency region. It is to be mentioned that in order to remove 60 Hz artifact, at the beginning, a digital notch filter is used and raw EEG signals are normalized to zero mean and unit variance. In the result section, effect of considering band-limited EEG signals with different band widths on mental task classification is presented.

### Feature extraction

A given frame of EEG data can be effectively modeled as the output of an AR system excited by white Gaussian noise [7, 8]. Considering the EEG signal as an output of a causal, stable, linear time-invariant AR system, it can be expressed as

1 $$\begin{aligned} x(n)=-\sum _{k=1}^{p}a_kx(n-k)+u(n), \end{aligned}$$

where *u*(*n*) is considered as white Gaussian noise input with zero mean and variance $\sigma _u^2$. Using the Yule-Walker equations, the AR parameters can be calculated as [15, 16]

2 $$\begin{aligned} r_x(m)= & {}\, -\sum _{k=1}^{p}a_kr_x(m-k)+\sigma _u^2\delta (m),\,\,m\ge 0\nonumber \\= & {}\, r_x(-m),\,\,m<0 \end{aligned}$$

where $r_x(m)$, the m-th lag of autocorrelation function (ACF) of *x*(*n*) with length *N*, can be termed as

3 $$\begin{aligned} r_x(m)=\frac{1}{N}\sum _{n=0}^{N-1-m}x(n)x(n+m),\,\,m\ge 0. \end{aligned}$$

**Fig. 2:**
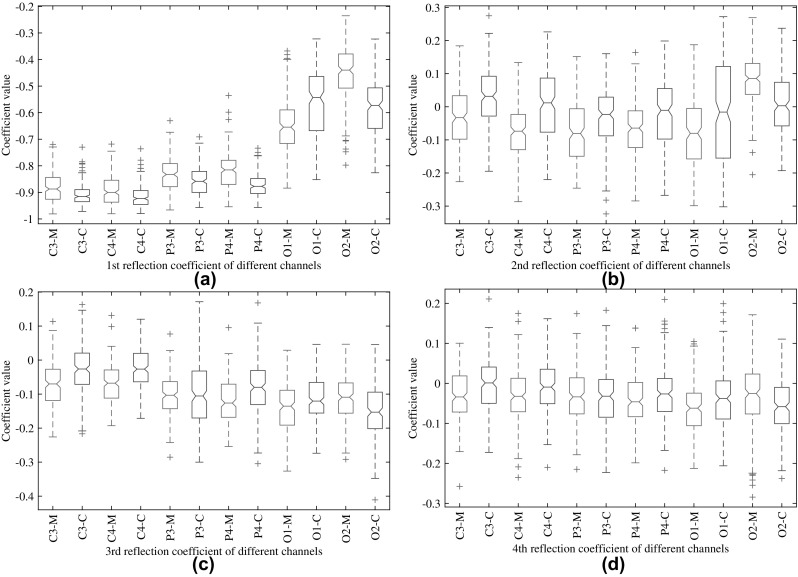
Statistical information of reflection coefficients of different channels obtained from subject 1 considering mathematical multiplication and visual counting task. **a**–**d** correspond to statistical information of 1st, 2nd 3rd and 4th reflection coefficients, respectively

AR parameters have already been used as features for EEG signal classification. For example, in [7], sixth-order AR parameters are used for EEG-based mental task classification. One major problem in using AR parameters as features is the wide range of variation in parameter values, which does not have any boundary. As an alternate to the AR parameters, in this paper, we propose to utilize reflection coefficients as representative features.

The *m*-th reflection coefficient computes the correlation between *x*(*n*) and $x(n-m)$ after filtering the intermediate observations from $x(n-1)$ to $x(n-m+1)$. It can be obtained directly from ACF values of given signal *x*(*n*) by utilizing the Levinson–Durbin recursion formulas as [17]

4 $$\begin{aligned} k_m=\frac{r_x(m)-\sum _{j=1}^{m-1}d_j^{(m-1)}r_x(m-j)}{E^{(m-1)}}, m=1,2,..,p \end{aligned}$$

where $d_j^{(m)}$ at the *m*-th iteration can be estimated as

5 $$\begin{aligned} d_j^{(m)}= & {}\, d_j^{(m-1)}-k_md_{(m-j)}^{(m-1)},1\le j\le {(m-1)}\nonumber \\ d_m^{(m)}= & {}\, k_m. \end{aligned}$$

Here, $E^{(m)}$ is the residual energy at *m*-th iteration and can be estimated as

6 $$\begin{aligned} E^{(m)}=\left( 1-k_m^2\right) E^{(m-1)},m\ge 1\,\hbox {and}\,E^{(0)}=r_x(0). \end{aligned}$$

In summary, use of reflection coefficients as features provides following advantages in comparison with the AR parameters:

1. As described above, the reflection coefficients can be directly obtained from autocorrelation values of given EEG data by utilizing simple recursive formula. Complicated AR parameter estimation method involving matrix inversion is not necessary for obtaining reflection coefficients.
2. One problem in AR parameter values is that there is no certain limit for the value of an AR parameter. A feature value without a specific bound may create problem in feature based classification problem. On the other hand, the value of a reflection coefficient $(k_m)$ is bounded for stable AR systems, which is $|k_m|< 1$. Given EEG data is modeled as the output of stable AR system.
3. It is found that the effect of different types of external noises, such as power line noise, load noise and muscle noise can cause less variation in reflection parameters in comparison with AR parameters. This may occur due to the process of computing reflection coefficients which involves a very few arithmetic operation using few ACF values in comparison with the case of AR parameter estimation. For example, to obtain first two reflection coefficients, only following operations are required.
  7 $$\begin{aligned} k_1 =  {}\, \frac{r_x(1)}{r_x(0)},~~ k_2 &= \frac{r_x(2)-k_1r_x(1)}{(1-k_1^2)r_x(0)} \nonumber \\ &=  {}\, \frac{1}{1-k_1^2}\left( \frac{r_x(2)}{r_x(0)}-k_1^2\right) . \end{aligned}$$

Thus, reflection coefficients acquired from the autocorrelation function of the EEG signal have the potential to form a distinctive feature vector for mental task classification. One major concern is the number of reflection coefficients to be computed for feature extraction. In fact, it is a common problem in the AR modeling of EEG signal to find the model order that is appropriate for the given data. Considering different model order will provide AR parameters those are completely different. However, with the increase in model order by one for a given signal, only the value of the last (highest order) reflection coefficient will be changed. Considering the size of the feature vector, only first few reflection coefficients can be chosen.

In order to investigate the number of reflection coefficients to be considered as feature, detailed statistical analysis on first few reflection coefficients is performed. As an example, EEG recordings of all the sessions of a particular subject performing mathematical multiplication (M) and visual counting (C) tasks are considered. After performing the necessary preprocessing, the reflection coefficients are extracted. In this case, four reflection coefficients are computed considering AR (4) system. In Fig. 2a, the boxplots of the first reflection coefficient indicating some statistical information, such as median, 25th and 75th percentile, and outliers are shown. There are twelve boxplots corresponding to six channel data with two different types of tasks, indicated with two different letters M and C after the channel label. To be more specific, C3-M and C3-C correspond to boxplots obtained from the EEG data of channel C3; first one refers to mathematical multiplication and the second one to visual counting tasks. The boxplots computed for other tasks are excluded here to avoid complicated presentation.

From Fig. 2, it is observed that 1st and 2nd reflection coefficients offer better discriminative features between two classes of mental tasks in comparison with other two reflection coefficients. For example, the statistical characteristics of the 3rd and 4th reflection coefficients in channel P3 exhibit poor discriminative feature, which may affect the performance of classification between two types of mental tasks. This statement is also reflected in nonparametric Wilcoxon rank sum test which is used to quantify the test of class separability in terms of medians. It tests whether data from two types of tasks come from identical continuous distributions with equal medians, against the alternative that they do not have equal medians. The probability values (p-value) under this null hypothesis are found 0.00012472, 0.00000058, 0.96163331 and 0.55842113, respectively, for four reflection coefficients considering channel P3. The high p-values observed for 3rd and 4th reflection coefficient indicate the failure to reject the null hypothesis, i.e., the data obtained from two tasks have almost similar medians reflecting low separability of the feature. On the contrary, very low p-values obtained in case of 1st and 2nd reflection coefficients indicate that the data obtained from two tasks does not come from identical distributions with almost equal medians. Hence, it is expected that the use of only first two reflection coefficients as feature can precisely preserve important discriminative information of the original signal patterns. For this purpose, it is adequate to consider second order AR modeling to obtain the proposed reflection coefficients. For an *l* channel EEG data, considering two reflection coefficients from each channel, the feature dimension will be 2*l*. As a result, it will also offer significant reduction in feature dimension. However, effect of varying the number of reflection coefficients on the classification performance is described in detail in the result section.

### Classification

Selecting an efficient classifier for the classification of EEG data into different groups plays an important role in obtaining satisfactory performance. However, if discriminative characteristics of different classes can be extracted, simple classifier may provide reasonable performance. Instead of directly utilizing the data or their statistics as feature, in the proposed method, classification is carried out on the features extracted from the data. Because of its wide acceptability and effectiveness in supervised classification, for the purpose of classifying the EEG signal, kernel-based SVM classifier is employed. The kernel-based approach converts the data from the original space to a new representative vector space, where it becomes easier to discriminate different classes more efficiently. The objective is to compute an *N* dimensional decision vector $\mathbf {w}= [w_1~w_2~\cdots ~w_N]^T$ for a training dataset which consists of *L* frames, where each *N* dimensional *i*-th frame ${\mathbf {x}}_{i} = x_{i} (n),n = 1,\cdots ,N$ is marked with a class label $y_i$. The decision vector is formed from a given discriminating function $f(\mathbf {x})=f(\mathbf {w}, \mathbf {x})$, which can effectively match with class label $y_i$ of the training dataset. In SVM, the training vectors ${\mathbf {x}}_{i}$ fulfill the following inequalities considering the threshold *b* for two class problem with two class values $+1$ and $-1$ [18]:

8 $$\begin{aligned} {\mathbf {w^T}} {\mathbf{x}}_{i}+b\ge & {} +1,\text{ for } \text{ all } \text{ positive } {\mathbf{x}}_{i}\nonumber \\ {\mathbf {w^T}} {\mathbf{x}}_{i}+b\le & {} -1,\text{ for } \text{ all } \text{ negative } {\mathbf{x}}_{i} \end{aligned}$$

For kernel-based classification approach, the discriminant function *f*(**x**) can be defined as [18]

9 $$\begin{aligned} f(\mathbf{x})=\sum _{i=1}^Lc_iK({\mathbf{x}}_{i},{\mathbf{x}})+b, \end{aligned}$$

where $c_i$ is an empirical value and kernel matrix **K** is given by

10 $$\begin{aligned} \mathbf {K}=\begin{bmatrix} K({\mathbf{x}}_{1},{\mathbf{x}}_{1})&K({\mathbf{x}}_{1},{\mathbf{x}}_{2})&\cdots&K({\mathbf{x}}_{1},{\mathbf{x}}_{L})\\ K({\mathbf{x}}_{2},{\mathbf{x}}_{1})&K({\mathbf{x}}_{2},{\mathbf{x}}_{2})&\cdots&K({\mathbf{x}}_{2},{\mathbf{x}}_{L}) \\ \vdots&\vdots&\cdots&\vdots \\ K({\mathbf{x}}_{L},{\mathbf{x}}_{1})&K({\mathbf{x}}_{L},{\mathbf{x}}_{2})&\cdots&K({\mathbf{x}}_{L},{\mathbf{x}}_{L}) \end{bmatrix} \end{aligned}$$

It is to be mentioned that the *i*, *j*-th element of the kernel matrix for both linear and nonlinear kernel function can be characterized as the inner product of the *i*-th and *j*-th training vectors.

In this paper, the effect of different kernel functions is investigated, and finally, quadratic kernel function is used in our proposed scheme for mental task classification. For the sake of evaluating the performance of extracted features, leave-one-out cross-validation scheme is applied, where each frame is tested one by one. During the testing of a frame, all the remaining frames are used for training. The overall accuracy is calculated based on the classification results obtained in all the frames.

## Simulation and results

In this section, performance of the proposed method is investigated considering classification accuracy obtained under different conditions, such as varying the feature dimension, utilizing different spectral bands for feature extraction and employing various types of kernel function in SVM classifier. Moreover, effect of channel selection on classification accuracy using the proposed method is analyzed. In this case, various spatial locations of EEG channels are taken into consideration. A comparative analysis on classification performance between the proposed method and some existing methods is also performed. In the proposed method, two reflection coefficients obtained from each channel are computed using (7) and used as discriminative feature to classify EEG signal. Unless otherwise specified, quadratic kernel of SVM classifier is employed in leave-one-out cross-validation manner to obtain classification accuracy. The classification task is carried out considering two types of mental tasks at a time, as conventionally done by other researchers [5, 6]. In this way, ten different combinations of these five types of tasks are plausible where each combination is denoted by two alphabets. For example, MC corresponds to combination of mathematical multiplication and visual counting tasks, BL corresponds to combination of baseline-resting and mental letter composing tasks etc. In what follows, detail results and analyses are presented.

### Effect of channel selection

**Fig. 3:**
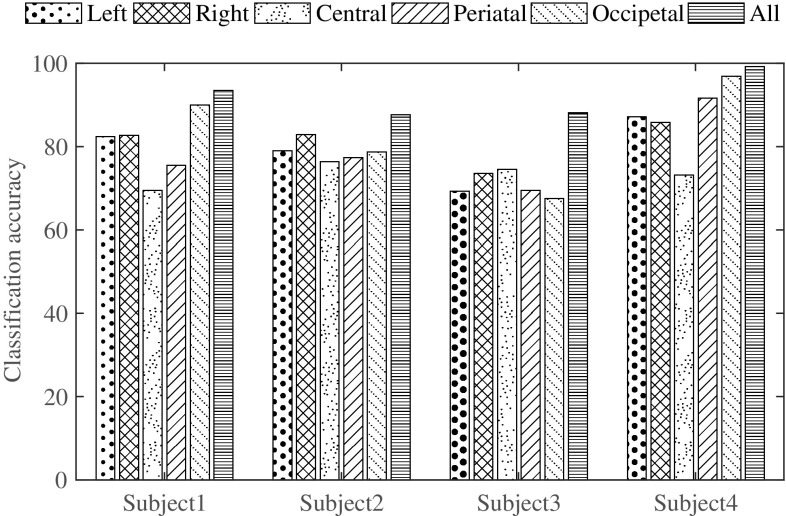
Effect of channel selection on classification accuracy for all four subjects in case of MC pair of tasks

In mental task classification-based applications, it is necessary to perform the testing phase within the frame rate in view of obtaining real-time classification results. For that purpose, channel selection can play a vital role to reduce feature size effectively, which in turn reduces the complexity as well as time involved in performing the classification task. In the given database, EEG signals are acquired from six locations, namely C3, P3, O1, C4, P4, and O2, where the first three channels are placed in the left hemisphere and the last three channels are placed in the right hemisphere of the skull. One possibility is to consider EEG channels located in any one of the two hemispheres. In that case, half of the channels will be reduced. Alternately, considering only central channels (C3, C4) or parietal channels (P3, P4) or occipital channels (O1, O2) can be used to investigate corresponding classification performance. In order to present the performance comparison between the cases where reduced number of channels and all channels are used, a sample experiment considering the mathematical multiplication and visual counting tasks for all four subjects is chosen. Classification accuracies obtained for four subjects using leave-one-out cross-validation technique are shown in Fig. 3.

It is observed that right hemisphere channels provide comparatively better accuracies with respect to that obtained by using the left hemisphere channels (except for subject 4). Moreover, a particular location of channels may provide better classification accuracy, but it varies from subject to subject. For example, for subject 1 and subject 4, better classification accuracy is achieved when channels from occipital locations are considered. However, in all cases, lower classification accuracy is obtained if the reduced number of channels are used instead of all channels. As a result, it is not possible to select any one particular choice of reduced number of channels to obtain acceptable classification performance in all subjects. Hence, in the proposed method, all channels are taken into account to perform the task of classification.

### Effect of feature dimension reduction using principal component analysis

**Fig. 4:**
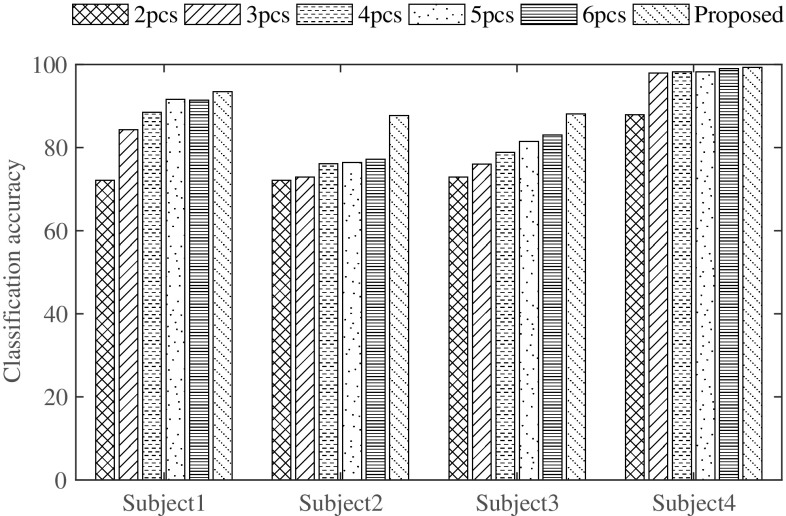
Effect of PCA on classification accuracy for all four subjects in case of MC pair of tasks

Feature dimension reduction using principal component analysis (PCA) may provide effective classification performance and reduced classification time. PCA is a statistical procedure that represents the original feature matrix in the principal component space with a view to producing a reduced number of principal components than the number of original features. For the purpose of investigation of varying the number of principal components, a sample experiment similar to that considered in the previous subsection is conducted. Here, the number of principal components is varied from 2 to 6 and different cases like using first two principal components (2pcs), first three principal components (3pcs) etc. for all four subjects are considered. Classification performance obtained by varying number of principal components are shown in Fig. 4.

It is observed that with the increase in number of principal components, classification accuracies obtained for different subjects increase. In case of subject 4, considering more than two principal components provide classification accuracies which are almost equal to that obtained by using all 12 features utilized in the proposed method. However, in all cases, classification accuracy obtained by using reduced number of principal components does not surpass accuracy that obtained by using all 12 features utilized in the proposed method. As a result, feature dimension reduction scheme using PCA is not taken into account to perform the task of classification in the proposed method.

### Effect of variation of number of reflection coefficients

**Fig. 5:**
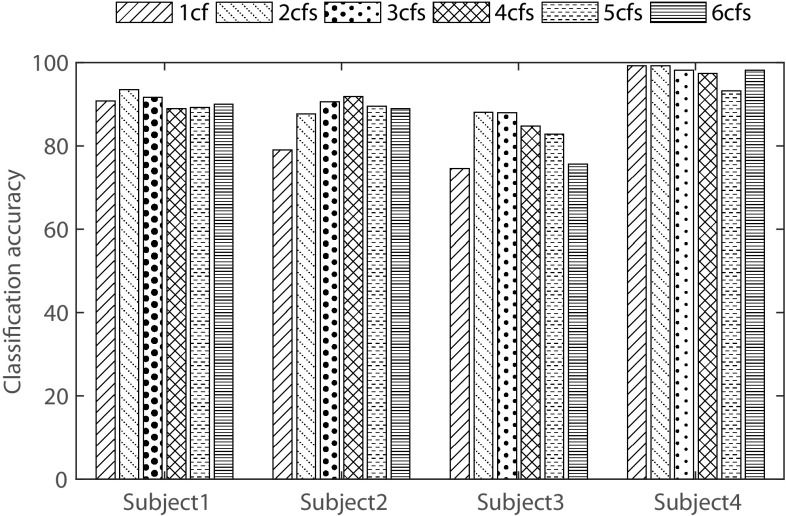
Effect of reflection coefficients variation on classification accuracy for all four subjects in case of MC pair of tasks

The number of reflection coefficients to be used in the feature matrix directly dictates the feature dimension. In general, lower order AR model is sufficient to represent EEG data. In that case, a few number of reflection coefficients, say less than six, are sufficient to consider as feature. With a view to investigate the effect of varying the number of reflection coefficients, a sample experiment similar to that considered in the previous subsections is chosen. Here, the number of reflection coefficients is varied from 1 to 6 and different cases like using the first coefficient (1cf), first two coefficients (2cfs) etc. for all four subjects are considered. In Fig. 5, classification performance obtained by varying number of reflection coefficients is presented.

It is found that increasing the number of reflection coefficients from one to two provides significantly increased accuracy (except in case of subject 4). It is very interesting that if the number of coefficients is taken more than two, the accuracy does not improve or even fall (except in case of subject 2). From this experiment, one may conclude to select first two or first three coefficients as feature. However, as stated earlier, reducing the overall feature dimension is always very essential for real-time computation. Hence, first two reflection coefficients are considered as proposed feature.

### Effect of frequency band selection

In different EEG signal analysis, most commonly band-limited signals are used considering conventional frequency bands, namely delta, theta, alpha, beta, and gamma [5]. Estimating reflection coefficients from a specific band-limited EEG signal may not be capable of providing representative characteristics. However, for the purpose of investigation, each band of EEG signal is separately generated by using narrow-band filters and first two reflection coefficients are estimated from the band-limited EEG signal. Classification performance for each band is separately computed. Moreover, various wide-band signals, such as $40-100$ Hz or $4-37$ Hz signals, are also taken in consideration and here also classification performance are computed considering the first two reflection coefficients.

**Fig. 6:**
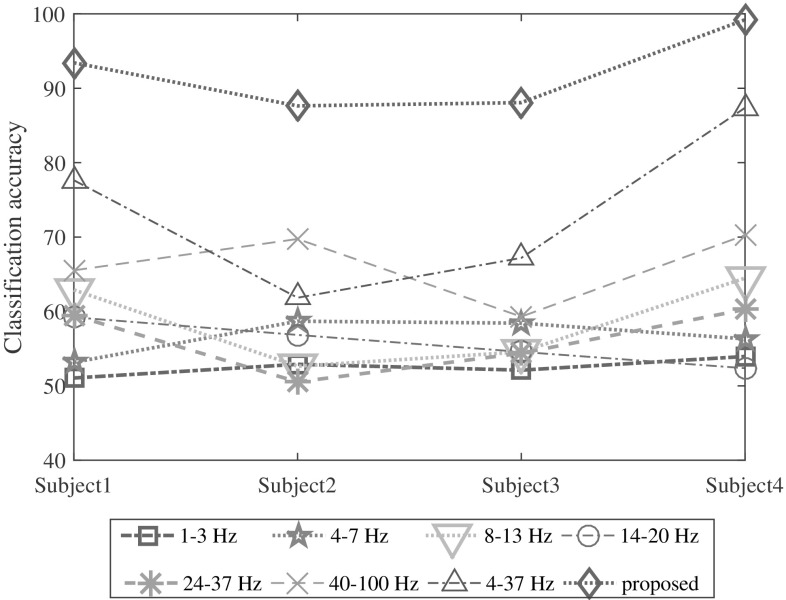
Effect of frequency band selection on classification accuracy for all four subjects in case of MC pair of tasks. (Color figure online)

The variation of classification performance for different band-limited EEG signals is demonstrated considering the similar experiment performed in the previous subsections. It is vividly observed from Fig. 6 that extracting features from different narrow-band EEG signals cannot provide satisfactory performance. However, considering wide-band EEG signals offer comparatively better performance than narrow-band EEG signals. In particular, without restricting the frequency band up to certain range, the best classification performance is achieved. That is why band limitation of the given EEG data is not adopted in this paper.

### Performance comparison with existing methods

The classification performance of the proposed method and that of the three available methods reported in [5, 6] is compared. Among these three methods, the first one utilizes power of spectral bands and asymmetry ratios from four bands (referred to as PAR4) and the second one also utilizes similar power and asymmetry ratios from five bands including the Gamma band (referred to as PAR5) as features. The third one introduces one additional band ($40-100$ Hz) along with the five bands utilized in third method and extracts power and asymmetry ratios as features (referred to as PAR6). For one pair of electrodes, the asymmetry ratio for each spectral band is computed as [5]

11 $$\begin{aligned} A(i,j)=\frac{P(i)-P(j)}{P(i)+P(j)} \end{aligned}$$

where two indices i and j are used to correspond electrode pairs placed in the left and right hemispheres, respectively. For example, *P*(*i*) corresponds to the spectral band power of the *i*-th electrode placed in the left hemisphere and *P*(*j*) corresponds to that obtained from the *j*-th electrode placed in the right hemisphere. Depending on the number of electrodes ($N_i$ and $N_j$) in each hemisphere, total $N_i\times N_j$ number of asymmetry ratios, denoted by *A*(*i*, *j*), can be computed for each spectral band. As a result, the feature dimension for PAR4, PAR5 and PAR6 method is ($N_b\times N_i\times N_j$ + $N_b\times l$) where $N_b$ denotes number of spectral band considered for these methods. On the contrary, as discussed before, considering two reflection coefficients from each channel, the feature dimension will be 2*l* for proposed method which is diminutive compare to PAR4, PAR5 and PAR6 method.

For the purpose of performance evaluation, leave-one-out cross-validation technique is carried out in all three existing methods. The SVM classifier considering linear (Ln), quadratic (Qd) and polynomial (Pl) kernel is employed in all three existing methods for the sake of fair comparison. As mentioned before, quadratic kernel of SVM classifier is employed in leave-one-out cross-validation manner to obtain classification accuracy for the proposed method. However, the effect of other kernels of SVM classifier on the classification performance obtained by proposed method is discussed exclusively in Sec. 4.7. In Tables 1, 2, 3 and 4, the classification accuracies obtained by using four different subjects are separately reported for PAR4, PAR5, PAR6 and proposed method. Moreover, average classification accuracy and standard deviation obtained from four subjects for different combination of tasks are listed in Table 5. It is found that the average classification accuracies obtained from different subjects are more than $89.16\%$ with reasonable amount of standard deviation for the proposed method. It is observed that the proposed feature extraction method outperforms other existing methods reported in this paper in terms of classification accuracy if quadratic kernel is considered to compute accuracy. For Subject 1 and Subject 3, it is observed that the proposed feature extraction method provides better classification accuracy irrespective of the kernel. However, in some combinations of mental tasks for Subject 2 and Subject 4, existing PAR6 method utilizing polynomial kernel offers competitive classification performance with respect to the proposed method, where a large feature dimension is required. For example, the classification accuracy of the proposed method obtained for Subject 2 is found higher for all but MR and CB combination of tasks where PAR6(Po) offers slightly better accuracy. In case of Subject 4, it is found that the average classification accuracy obtained for the proposed method is very close to PAR6(Po) despite having a very smaller feature dimension. In each reported existing method, it is observed that for various combination of mental tasks, classification accuracy varies a lot. For example, in PAR4(Li) method, the standard deviation of classification accuracies for various subjects are found $10.96\%$, $4.27\%$, $4.79\%$ and $7.92\%$ compared to $4.74\%$, $2.75\%$, $3.10\%$ and $5.80\%$ of the proposed method. It is found that the classification performance obtained by the proposed method varies from subject to subject, but not at a very large scale. It is clearly observed that the proposed method offers consistently satisfactory classification accuracy in most cases irrespective of subjects and combination of mental tasks.

**Table 1:** Overall classification accuracy obtained for subject 1

Task	PAR4(Li)	PAR4(Qu)	PAR4(Po)	PAR5(Li)	PAR5(Qu)	PAR5(Po)	PAR6(Li)	PAR6(Qu)	PAR6(Po)	Proposed
	[5]	[5]	[5]	[5]	[5]	[5]	[6]	[6]	[6]	
MC	88.16	79.21	88.42	88.42	78.68	88.95	92.37	82.37	91.58	93.42
MB	82.63	71.32	82.11	82.89	73.16	83.95	84.47	78.68	87.37	92.63
ML	90.53	72.37	86.05	90.53	71.32	87.37	93.95	82.11	90.26	96.05
MR	93.42	80.53	89.47	92.37	81.32	91.05	96.05	87.37	93.95	97.63
CB	72.37	65.53	72.37	74.74	68.42	78.95	83.42	80.26	82.63	91.05
CL	64.21	63.16	65.26	65.79	65.53	69.74	69.74	76.32	77.11	82.11
CR	72.11	68.42	71.58	71.05	71.32	74.74	73.42	77.63	80.26	87.63
BL	65.79	64.47	69.74	63.68	68.68	71.05	75.79	81.32	82.89	88.95
BR	86.05	77.89	83.42	85.00	78.16	86.58	90.00	86.05	92.37	94.21
LR	68.42	67.63	70.26	72.11	71.05	77.37	75.53	77.89	81.84	86.58
Avg	78.37	71.05	77.87	78.66	72.76	80.97	83.47	81.00	86.03	91.03
Std. dev	10.96	6.32	8.91	10.48	5.08	7.65	9.44	3.62	5.82	4.74

**Table 2:** Overall classification accuracy obtained for subject 2

Task	PAR4(Li)	PAR4(Qu)	PAR4(Po)	PAR5(Li)	PAR5(Qu)	PAR5(Po)	PAR6(Li)	PAR6(Qu)	PAR6(Po)	Proposed
	[5]	[5]	[5]	[5]	[5]	[5]	[6]	[6]	[6]	
MC	68.42	66.58	69.47	70.79	73.95	76.58	76.84	81.05	83.42	87.63
MB	76.05	70.53	78.95	82.37	79.47	86.32	86.32	85.79	91.05	94.47
ML	67.37	66.32	70.53	77.11	78.16	83.42	83.42	85.79	87.63	88.95
MR	66.32	62.89	71.32	71.84	74.74	79.21	75.53	85.53	90.00	87.89
CB	71.84	72.11	74.74	79.47	79.47	80.53	85.00	90.79	92.11	90.79
CL	65.53	60.26	64.74	71.58	71.05	75.53	88.95	85.26	89.47	94.74
CR	59.47	66.58	68.68	64.47	76.05	73.42	82.63	84.47	87.11	90.79
BL	68.68	66.84	71.84	76.84	74.74	79.47	84.74	84.47	86.84	91.32
BR	69.21	64.74	76.05	72.11	75.26	79.74	78.95	83.68	86.05	87.11
LR	68.42	67.11	71.58	70.00	70.53	80.79	76.84	82.89	84.21	88.16
Avg	68.13	66.39	71.79	73.66	75.34	79.50	81.92	84.97	87.79	90.18
Std. dev	4.27	3.38	4.01	5.25	3.11	3.74	4.60	2.53	2.85	2.75

**Table 3:** Overall classification accuracy obtained for subject 3

Task	PAR4(Li)	PAR4(Qu)	PAR4(Po)	PAR5(Li)	PAR5(Qu)	PAR5(Po)	PAR6(Li)	PAR6(Qu)	PAR6(Po)	Proposed
	[5]	[5]	[5]	[5]	[5]	[5]	[6]	[6]	[6]	
MC	63.51	61.58	68.42	66.84	66.49	74.91	69.47	74.04	79.82	88.07
MB	69.82	66.84	73.16	71.05	71.75	74.56	74.91	81.75	81.40	87.89
ML	69.65	67.89	71.58	68.25	70.18	74.39	75.96	77.19	80.70	90.53
MR	73.68	67.19	74.74	79.30	72.46	79.47	81.93	83.33	87.89	93.51
CB	67.02	61.23	70.53	66.49	63.68	72.98	72.46	71.75	81.05	85.61
CL	72.11	64.21	72.81	71.93	66.84	77.19	79.12	75.09	80.35	86.49
CR	71.40	61.23	68.60	73.16	67.54	75.44	76.49	78.95	81.40	91.40
BL	71.23	64.91	74.39	73.51	64.74	74.56	78.07	74.91	84.21	86.67
BR	71.05	68.95	73.86	69.30	70.53	75.96	80.18	77.19	84.21	87.02
LR	82.11	69.82	77.89	83.68	76.67	83.33	89.47	78.95	87.54	94.39
Avg	71.16	65.39	72.60	72.35	69.09	76.28	77.81	77.32	82.86	89.16
Std. dev	4.79	3.24	2.92	5.49	3.96	3.05	5.50	3.55	2.96	3.10

**Table 4:** Overall classification accuracy obtained for subject 4

Task	PAR4(Li)	PAR4(Qu)	PAR4(Po)	PAR5(Li)	PAR5(Qu)	PAR5(Po)	PAR6(Li)	PAR6(Qu)	PAR6(Po)	Proposed
	[5]	[5]	[5]	[5]	[5]	[5]	[6]	[6]	[6]	
MC	92.11	76.05	83.95	93.68	86.32	90.53	98.42	93.95	97.63	99.21
MB	87.89	80.53	86.84	92.11	81.58	90.53	95.00	91.05	94.74	97.63
ML	89.47	77.11	86.05	89.47	84.47	88.42	93.42	90.26	92.89	95.26
MR	89.21	74.21	84.21	90.53	79.21	88.95	95.00	88.95	93.95	92.63
CB	78.42	68.68	81.58	78.95	76.05	82.37	84.47	80.26	86.58	91.84
CL	79.47	74.47	78.16	81.84	80.00	81.58	85.26	83.95	87.63	87.63
CR	86.32	83.68	88.68	87.89	85.26	92.89	93.42	90.53	96.32	95.53
BL	68.95	62.37	68.16	70.53	71.05	77.63	73.95	75.53	83.95	79.47
BR	93.16	87.63	94.47	93.68	87.37	95.26	95.00	91.58	97.11	96.84
LR	93.68	88.95	94.47	93.68	91.05	96.05	93.42	91.05	97.37	91.05
Avg	85.87	77.37	84.66	87.24	82.24	88.42	90.74	87.71	92.82	92.71
Std. dev	7.92	8.23	7.75	7.76	5.90	6.09	7.35	5.87	4.99	5.80

**Table 5:** Average classification accuracy and standard deviation obtained from four subjects for different combination of tasks

Combination of tasks	MC	MB	ML	MR	CB	CL	CR	BL	BR	LR
Avg	92.08	93.16	92.7	92.92	89.82	87.74	91.34	86.6	91.29	90.04
Std. dev	5.43	4.07	3.49	4	2.84	5.24	3.24	5.12	5	3.44

### Physical insight on classification performance variation

It is observed in Tables 1, 2, 3, 4 and 5, comparatively better classification accuracy is obtained while considering MC, MB, ML and MR tasks than other six combinations of tasks. The important factor here is that in all those four cases, multiplication task is involved, which is the only mental arithmetic task among five different tasks considered in this experiment. It is expected that being an arithmetic task, multiplication involves more complexity in comparison with other four mental tasks, namely geometrical figure rotation, letter composing, counting and baseline-resting. As a result, characteristics of the EEG signals are expected to be significantly different in case of multiplication task and thus better classification performance is achieved whenever one of the two tasks to be classified is a multiplication task.

It is also observed that larger the degree of variation between the nature of two mental tasks, better the classification performance. For example, among four mental tasks (R, B, L, C), in case of geometrical figure rotation (R), subjects are trained to visualize a complex rotating block, which is comparatively difficult than other three mental tasks. As a result, it is found that the classification performance for the tasks RL, RB, and RC is better than the tasks BL, BC, and LC. Obviously, the above observations may vary subject to subject since a particular task may not be equally difficult to every subject.

### Effect of kernel in SVM classifier

**Fig. 7:**
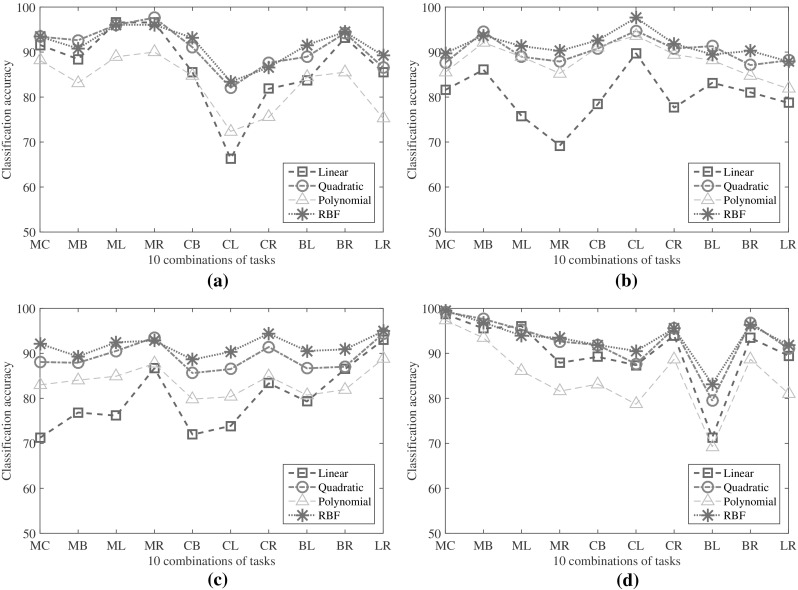
Classification accuracy obtained from four subjects considering different kernels in SVM classifier. **a**–**d** correspond to classification accuracy obtained from 1st, 2nd, 3rd and 4th subject considering different kernels respectively. (Color figure online)

Classification results of the proposed method presented in Tables 1, 2, 3 and 4 are obtained by using quadratic kernel in the SVM classifier. However, the effect of using different kernels in SVM classifier on overall classification performance is also investigated. In order to demonstrate the performance variation due to change in kernels, four widely used kernels are considered, namely linear, quadratic, polynomial, and radial basis function (RBF). Similar to Tables 1, 2, 3 and 4, considering 10 different combinations of tasks, in Fig. 7, classification accuracies obtained by using four different kernels are shown.

It is found that the classification performances of quadratic and RBF kernels are consistently better in comparison with those obtained by linear and polynomial kernels. Moreover, classification accuracy varies for different tasks in case of linear and polynomial kernels. Among quadratic and RBF kernels, since the first one provides better performance irrespective of feature dimension, in the results presented in Tables 1, 2, 3 and 4, quadratic kernel is chosen to classify EEG signals for the sake of comparing the proposed method with existing methods, which utilize larger feature dimension.

### Computation time

Average computational time is measured to extract features from one test signal for four methods namely PAR4, PAR5, PAR6 and proposed method. The whole process of computation is performed using Intel(R) Core(TM) i3-4130 processor with 3.40 GHz clock speed and 4 GB ram. The feature dimension and the feature extraction time for four methods are listed in Table 6.

**Table 6:** Feature dimension and average time for feature extraction

Feature characteristics	PAR4	PAR5	PAR6	Proposed
Feature dimension	60	75	90	12
Average time (ms)	41.70	51.64	61.94	1.52

It is found that the proposed method uses a very small computation time for feature extraction compare to other three methods. The reason for such a very small computation time for the proposed method is the feature dimension. In case of proposed method, the feature dimension is only $2\times 6=12$, while in case of PAR4, PAR5 and PAR6, it is $4\times 3\times 3+4\times 6=60$, $5\times 3\times 3+5\times 6=75$ and $6\times 3\times 3+6\times 6=90$ respectively.

## Conclusion

In the proposed mental task classification scheme, instead of conventional AR parameters, reflection coefficients of EEG data are utilized, which offers some major advantages, such as noise robustness, variation of values within a certain boundary and ease of computing via recursive relations. As a matter of fact, a quite satisfactory performance using very low feature dimension is achieved. It is observed that increase in feature dimension by considering more reflection coefficients not necessarily provides better performance and thus only two coefficients from each channel are found sufficient. In addition, it is found that frequency band limitation is not necessary in the proposed scheme for obtaining consistent estimate of reflection coefficients, and thus available full band EEG signal is utilized. Effect of channel selection is also investigated, and it is observed that for some subjects, a competitive classification performance may be obtained by considering only some specific channels. However, considering all channels provide the best classification performance irrespective of the task or the subject. It is observed that the proposed feature extraction method consistently offers better classification accuracy compared to various available methods reported in this paper despite having a very small feature dimension. The high classification accuracy and low standard deviation obtained from various combination of tasks indicate high within class compactness and between class separability of the proposed feature extraction scheme. Besides improving classification accuracy, the biological reasons behind obtaining variation in classification accuracies for different tasks are also investigated in this paper. Finally, it is shown that the proposed scheme offers very low computational time for feature extraction and classification. Results obtained from various types of investigation verify that the proposed mental task classification scheme is capable of classifying EEG signals with high classification accuracy and very low computational time.
